# Modeling and Analysis of a Three-Terminal-Memristor-Based Conservative Chaotic System

**DOI:** 10.3390/e23010071

**Published:** 2021-01-04

**Authors:** Ze Wang, Guoyuan Qi

**Affiliations:** Tianjin Key Laboratory of Advanced Technology of Electrical Engineering and Energy, Tiangong University, Tianjin 300387, China; 1830041172@tiangong.edu.cn

**Keywords:** three-terminal memristor, non-Hamiltonian conservative chaotic system, conservative chaos, analog circuit

## Abstract

In this paper, a three-terminal memristor is constructed and studied through changing dual-port output instead of one-port. A new conservative memristor-based chaotic system is built by embedding this three-terminal memristor into a newly proposed four-dimensional (4D) Euler equation. The generalized Hamiltonian energy function has been given, and it is composed of conservative and non-conservative parts of the Hamiltonian. The Hamiltonian of the Euler equation remains constant, while the three-terminal memristor’s Hamiltonian is mutative, causing non-conservation in energy. Through proof, only centers or saddles equilibria exist, which meets the definition of the conservative system. A non-Hamiltonian conservative chaotic system is proposed. The Hamiltonian of the conservative part determines whether the system can produce chaos or not. The non-conservative part affects the dynamic of the system based on the conservative part. The chaotic and quasiperiodic orbits are generated when the system has different Hamiltonian levels. Lyapunov exponent (*LE*), Poincaré map, bifurcation and Hamiltonian diagrams are used to analyze the dynamical behavior of the non-Hamiltonian conservative chaotic system. The frequency and initial values of the system have an extensive variable range. Through the mechanism adjustment, instead of trial-and-error, the maximum *LE* of the system can even reach an incredible value of 963. An analog circuit is implemented to verify the existence of the non-Hamiltonian conservative chaotic system, which overcomes the challenge that a little bias will lead to the disappearance of conservative chaos.

## 1. Introduction

Since the HP laboratory [1] confirmed memristors’ physical existence in 2008, the memristors [2] have received extensive attention from the academic community. Memristor [3] is a kind of nonlinear resistor with memory function. The memristor has been investigated and applied in non-volatile memory [3], artificial neural network [4], confidential communication [5], analog circuit [6], an artificial intelligence computer [4,6], biological behavior simulation [7], etc., showing great potential. 

Although two-terminal memristors have proved the basic principle of neurons, the synapses of one neuron are far more than one, so it is necessary to study multi-terminal memristors. The three-terminal Widrow-Hoff memristor [8] has carried out this kind of attempt by adding a control terminal to realize a three-terminal chemical memristor. A new floating gate silicon MOS (MOSFET) transistor [9] was similarly proposed, and Lai proposed field-effect transistors with nano ionic gates [10]. Mouttet proposed the basic definition of the three-terminal memristor [11] based on the two-terminal memristor passive nonlinear system [12] by Chua. Recently, using monolayer molybdenum disulfide, three-terminal [13], six-terminal, or even more synapses’ memristors were realized.

Chaos exists in mathematical models [14] and other aspects such as the macroeconomic model [15], the breaking of topological supersymmetry [16], etc. Chaotic systems are divided into the conservative system and dissipative system [17]. The Lyapunov dimensions of dissipative chaotic systems are fractional; for instance, if the system’s full dimension is three, the Lyapunov dimension is slightly higher than two. The divergence of the dissipative chaotic system is less than zero leading to the phase volume converging to zero with the exponential rate of the divergence. Hence, the passing trajectories of the dissipative system are not ergodic in the 3D space as the chaotic attractor looks like, but occupies zero space of the fractional dimension. The system orbits cannot traverse the entire space given by the initial value, and even most of the spatial range cannot be experienced. The poor ergodicity caused by the fractional dimension is the disadvantage in chaos-based encryption. It is easy to obtain exhaustive attacks when used in encryption systems [17,18]. However, the conservative chaos has a full dimension in phase volume, and encryption based on conservative systems is better than dissipative systems in anti-attack [17,18,19]. Constructing a memristor-based conservative chaotic system is helpful to the encryption of information and provides better security about information-theory.

A conservative chaotic system is a system in which the phase volume space remains unchanged [17,18,19], so the dimension is an integer [20], and the orbit can traverse and occupy the entire space given by the initial value. Compared with dissipative chaotic systems, conservative chaotic systems are scarce, and conservative chaos can be divided into two types: Hamiltonian conservative (energy conservative) and phase volume conservative [17]. Recently, Qi [17,18] first established the four-dimensional (4D) Euler equations. The 4D Euler equation modeling is essential in mathematics, rigid-body dynamics, and the structure of symplectic manifolds and fluid dynamics [17,18]. Based on this, a 4D conservative chaotic system was constructed, which is strictly conservative chaos. 

Nowadays, most of the research on memristors is based on dissipative chaos [7,14]. There are two research routes of two-terminal memristor chaotic systems based on dissipative system: (1) The memristor is used to replace nonlinear components such as the Chua circuits [14,21], oscillator circuits [22], etc. The hidden attractor, multistability [23], hyperchaotic and fractional-order form [24] were proposed. (2) The memristor is used as a feedback term to couple into a neuron model such as the Hindmarsh-Rose (HR) neuron model [7], etc. Some recent research methods on memristors, such as Chua’s periodic table [16,25,26] and the multidimensional scaling [27], have not explained the causes of chaos from the perspective of Hamiltonian energy.

Only a few two-terminal memristor systems are driven by piecewise function [28] or sine function [29,30] to obtain conservative chaotic systems. However, the values of positive Lyapunov exponent (*LE*) of these systems in [28,29,30] are too small. The system [29] just satisfies that the sum of *LE*s is zero, but the system does not analytically meet zero divergence requirements. More importantly, to our best knowledge, no conservative chaos based on three-terminal memristor has been studied. A memristor-based conservative chaotic system is more complicated with more parameters, which increases the keyspace of the chaos generator. Besides, a memristor-based conservative chaotic system with a high positive Lyapunov exponent is necessary for chaos-based encryption to generate the pseudo-random number.

We consider designing a conservative three-terminal memristor chaotic system based on the 4D Euler equation given in [18]. The 4D Euler equation is a very delicate ordinary differential equation based on mathematics, which is strictly conservative in both energy and divergence, but it just produces the periodic orbit instead of the chaos. By coupling the Euler equation with a three-terminal memristor, the energy conservation is broken, but the phase-volume conservation (divergence being zero) is still kept. Therefore, this paper constructs a three-terminal-memristor-based conservative chaotic system, and gives its energy function, pointing out the cause of its chaos. A large positive Lyapunov exponent is produced, which is advantageous over existing dissipative chaos and conservative chaos. An analog circuit is designed to prove the theory’s feasibility, and verify the existence of the non-Hamiltonian conservative chaotic system, which overcomes the challenge that a little bias will lead to the disappearance of conservative chaos.

This paper is organized as follows: Section 2 proposes the three-terminal memristor and constructs the circuit to implement it. Section 3 proposes the conservative chaotic system based on a three-terminal memristor from a strict conservative system [18]. Section 4 gives the characteristics of equilibria of the three-terminal memristor conservative system. Section 5 gives the dynamical analysis, and the cause of dynamical changing of the system in different levels and the impact of Hamiltonian on the system are investigated. Analog circuit implementation is provided in Section 6. Section 7 summarizes the paper.

## 2. Modeling of Three-Terminal Memristor

The memristor predicted by Chua is a two-port device [31]. It also has three significant features: the hysteresis loop passes the origin, the hysteresis loop is the shape of eight, and the area of the hysteresis curve decreases with increasing signal frequency. Here, we choose the cubic smooth memductance nonlinearity model [14,32]. The memristor model is described in the following form

(1) $$\begin{array}{l} \dot{\varphi }=v,\\ W(\varphi )=\alpha +\beta \varphi^{2},\\ i=W(\varphi )v=(\alpha +\beta \varphi^{2})v. \end{array}$$

According to the magnetic controlled three-terminal memristor model proposed in the memristive systems analysis [11], the following results are obtained

(2) $$\begin{array}{l} \frac{dw}{dt}=f\left(w,v_{g},v_{d}\right),\\ i_{g}=g\left(w,v_{g},v_{d}\right),\\ i_{d}=h\left(w,v_{g},v_{d}\right). \end{array}$$

Here $v_{g},v_{d}$ represent the input voltages, $i_{g},i_{d}$ represent the output currents, w donates an n-dimensional state variable of the system, g,h are defined as continuous function, and f is an n-dimensional continuous function. The memristor model in Equation (1) is appropriately deformed. By changing it to the model of single-port input and dual-port output, the model is transformed as

(3) $$\begin{array}{l} \frac{d\varphi }{dt}=f\left(w,v_{1},v_{2}\right)=v_{in}=v_{1}v_{2},\\ i_{1}=g\left(w,v_{1},v_{2}\right)=W(\varphi )v_{1}=(\alpha +\beta \varphi^{2})v_{1},\\ i_{2}=h\left(w,v_{1},v_{2}\right)=W(\varphi )v_{2}=(\alpha +\beta \varphi^{2})v_{2}. \end{array}$$

where $v_{in}=v_{1}v_{2}$ is the input voltage of the model, φ is the magnetic flux that controls the state of the model, W(φ) represents the memductance, and $i_{1},i_{2}$ are the two current outputs.

**Figure 1:**
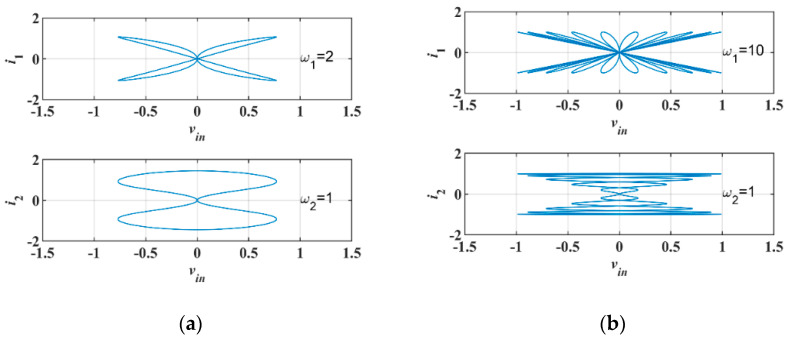
I-V curves with different frequency inputs: (**a**) $\omega_{1}=2,\;\omega_{2}=1$**,** (**b**) $\omega_{1}=10,\;\omega_{2}=1$.

**Figure 2:**
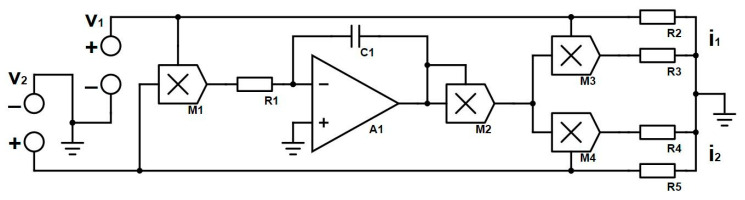
Circuit implementation of the three-terminal memristor.

To verify the feature of the model Equation (3), we used Matlab for numerical simulation, as shown in Figure 1, The product of $v_{1}=A_{1}\sin(\omega_{1}t)$ and $v_{2}=A_{2}\sin(\omega_{2}t)$ is the input of this device with parameters $A_{1}=A_{2}=\omega_{2}=1$, and $\omega_{1}=2$ in Figure 1a and $\omega_{1}=10$ Figure 1b. We found that the hysteresis curve does not converge to a single-valued function, but a multi-valued resistance with the frequency increasing, which means this model has a complex resistance value. The three-terminal memristor can be implemented by the circuit shown in Figure 2.

In Figure 2, $v_{1},v_{2}$ are input voltages of the model, $M_{1}$ to $M_{4}$ multipliers, $R_{1}$ to $R_{5}$ resistors, $C_{1}$ a capacitor, and $i_{1},i_{2}$ output currents, respectively. The analytical mathematical model is given as

(4) $$\begin{array}{l} i_{1}=\frac{v_{1}}{R_{2}}+\frac{{\left(\int v_{1}v_{2}dt\right)}^{2}v_{1}}{R_{1}^{2}C_{1}^{2}R_{3}},\\ i_{2}=\frac{v_{2}}{R_{5}}+\frac{{\left(\int v_{1}v_{2}dt\right)}^{2}v_{2}}{R_{1}^{2}C_{1}^{2}R_{4}}. \end{array}$$

**Figure 3:**
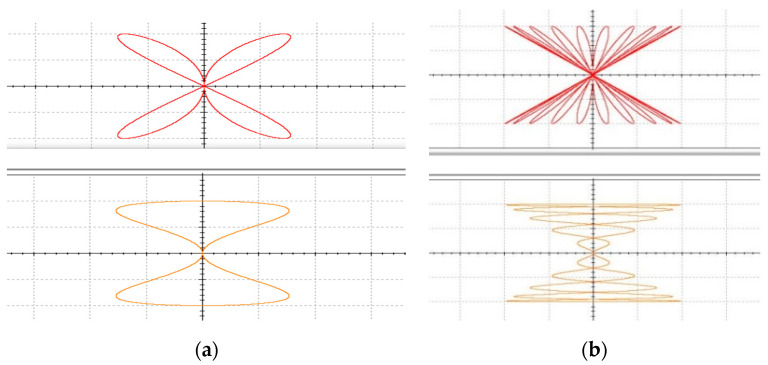
I-V curves of Multisim simulation circuit (**a**) $\omega_{1}=2,\;\omega_{2}=1$ (**b**) $\omega_{1}=10,\;\omega_{2}=1$.

$R_{1},C_{1}$ form the integrated circuit, and the remaining four resistors match the output coefficients. Using Multisim to simulate the model, input voltages are consistent with Matlab numerical simulation. The circuit simulation results received are shown in Figure 3.

It was found that the simulation results of the circuit model are consistent with the simulation of the mathematical model, so the model can be used to build the actual hardware circuit.

## 3. Modeling of Conservative Chaotic System Based on Three-Terminal Memristor

Qi proposed a 4D Euler rigid body equation with a Hamiltonian vector field form [18]

(5) $$\dot{x}=J(x)\nabla H(x),$$

where

(6) $$J(x)=\left[\begin{array}{cccc} 0 & -x_{3} & x_{2} & 0\\ x_{3} & 0 & -x_{1}-x_{4} & x_{3}\\ -x_{2} & x_{1}+x_{4} & 0 & -x_{2}\\ 0 & -x_{3} & x_{2} & 0 \end{array}\right],$$

with

(7) $$H(x)=\frac{1}{2}(\pi_{1}x_{1}^{2}+\pi_{2}x_{2}^{2}+\pi_{3}x_{3}^{2}+\pi_{4}x_{4}^{2}).$$

The system can be written as

(8) $$\begin{array}{l} {\dot{x}}_{1}=(\pi_{3}-\pi_{2})x_{2}x_{3},\\ {\dot{x}}_{2}=(\pi_{1}-\pi_{3})x_{1}x_{3}+(\pi_{4}-\pi_{3})x_{3}x_{4},\\ {\dot{x}}_{3}=(\pi_{2}-\pi_{1})x_{1}x_{2}+(\pi_{2}-\pi_{4})x_{2}x_{4},\\ {\dot{x}}_{4}=(\pi_{3}-\pi_{2})x_{2}x_{3}. \end{array}$$

The divergence of the 4D Euler equation is

(9) $$\nabla \cdot \dot{x}=\sum_{i=1}^{4}\frac{\partial f_{x_{i}}}{\partial x_{i}}=0.$$

Therefore, Equation (8) is a phase-volume conservative system. Because J(x) in Equation (6) is a skew-symmetric matrix, we have

(10) $$\dot{H}=\nabla H{\left(x\right)}^{T}J(x)\nabla H(x)=0.$$

**Figure 4:**
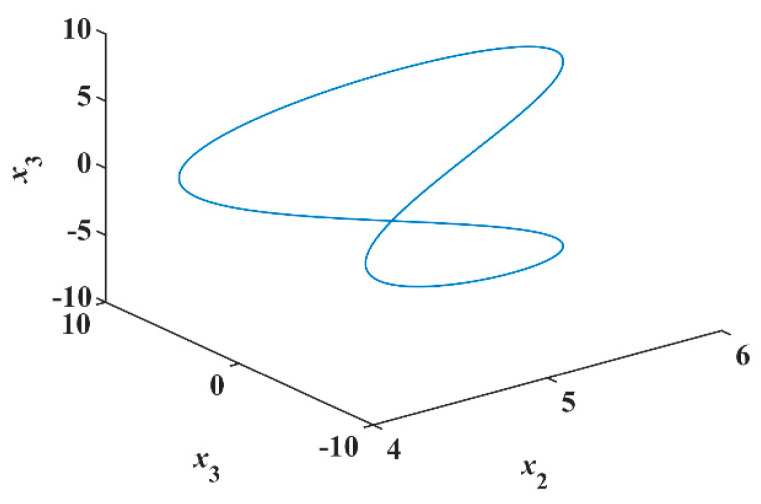
Periodic orbit of the system of Equation (8).

Thus, the system of Equation (8) is a Hamiltonian conservative system. Therefore, it preserves both the phase-volume and Hamiltonian. Using Matlab for numerical simulation, take parameters ${\left[\pi_{1},\;\pi_{2},\;\pi_{3},\;\pi_{4}\right]}^{T}={\left[2,\;3,\;4,\;5\right]}^{T}$, initial conditions ${\left[x_{10},\;x_{20},\;x_{30},\;x_{40}\right]}^{T}={\left[5,\;5,\;-5,\;-5\right]}^{T}$, and sampling time T=0.001 s. Since the conservation of both phase-volume and Hamiltonian, this system only produces periodic orbit [17,18,19], as shown in Figure 4.

The 4-D Hamiltonian conservative system only produces a periodic orbit because of the conservation of both phase volume and Hamiltonian energy. Does it generate conservative chaos by breaking one of the conservations, like the Hamiltonian energy? Qi [17] proposed a Hamiltonian conservative chaotic system by changing the Casimir conservation and keeping the Hamiltonian constant. Only one parameter was changed. So far, to our best knowledge, no memristor has been applied in all the conservative chaotic systems generation.

To generate chaos, we should break the conservation of Hamiltonian by adding a pair of constants c and $\left(\pi_{4}/\pi_{1}\right)c$ in the symplectic matrix J(x) in Equation (6), and then Equation (5) can be written as

(11) $$\dot{x}=J_{c}(x)\nabla H(x),$$

where

(12) $$J_{c}(x)=\left[\begin{array}{cccc} 0 & -x_{3} & x_{2} & c\\ x_{3} & 0 & -x_{1}-x_{4} & x_{3}\\ -x_{2} & x_{1}+x_{4} & 0 & -x_{2}\\ -\frac{\pi_{4}}{\pi_{1}}c & -x_{3} & x_{2} & 0 \end{array}\right],$$

For simplification, the system parameters are fixed as

(13) $${\left[\pi_{1},\;\pi_{2},\;\pi_{3},\;\pi_{4}\right]}^{T}={\left[2,\;3,\;4,\;5\right]}^{T}.$$

Then Equation (11) becomes

(14) $$\begin{array}{l} {\dot{x}}_{1}=x_{2}x_{3}+5cx_{4},\\ {\dot{x}}_{2}=-2x_{1}x_{3}+x_{3}x_{4},\\ {\dot{x}}_{3}=x_{1}x_{2}-2x_{2}x_{4},\\ {\dot{x}}_{4}=x_{2}x_{3}-5cx_{1}. \end{array}$$

with

(15) $$H(x)=\frac{1}{2}(2x_{1}^{2}+3x_{2}^{2}+4x_{3}^{2}+5x_{4}^{2}).$$

Because the main diagonal of Equation (12) is zero as Equation (6), the phase volume is still conservative. 

However, for the Hamiltonian energy, we have

(16) $$\dot{H}=\nabla H{\left(x\right)}^{T}J_{c}(x)\nabla H(x)=c\pi_{4}\left(\pi_{1}-\pi_{4}\right)x_{1}x_{4}=-15cx_{1}x_{4}.$$

**Figure 5:**
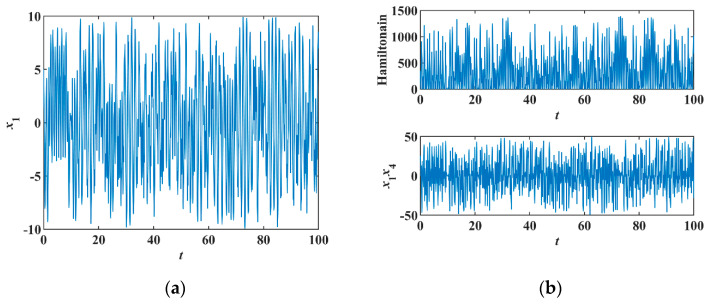
Chaotic orbits of system Equation (11), (**a**) time series of $x_{1}$, (**b**) time series of $x_{1}x_{4}$ and Hamiltonian.

Thus, the system is non-conservative in Hamiltonian energy but conservative in phase volume. Setting initial conditions ${\left[x_{10},\;x_{20},\;x_{30},\;x_{40}\right]}^{T}={\left[5\;,\;5\;,\;-5\;,\;-5\right]}^{T}$, constant c=1 and sampling time T=0.001 s, this system produces chaotic orbits [Figure 5a]; therefore, the Hamiltonian of Equation (12) is non-conservative because the positive and negative changes of $x_{1}x_{4}$ [Figure 5b].

When the three-terminal memristor is coupled with the 4D Euler equation, can chaos be generated using a similar way? The memristor model is regarded as a device inserted in the 4D rigid body to replace 5c and −5c. To get the input of the memristor, we added another variable $x_{5}$ as the input of the memristor. Thus, the memristor is $\alpha +\beta x_{5}^{2}$, and the new system is described as

(17) $$\begin{array}{l} {\dot{x}}_{1}=x_{2}x_{3}+\gamma (\alpha +\beta x_{5}^{2})x_{4},\\ {\dot{x}}_{2}=-2x_{1}x_{3}+x_{3}x_{4},\\ {\dot{x}}_{3}=x_{1}x_{2}-2x_{2}x_{4},\\ {\dot{x}}_{4}=x_{2}x_{3}-\gamma (\alpha +\beta x_{5}^{2})x_{1},\\ {\dot{x}}_{5}=x_{1}x_{4}. \end{array}$$

Here γ is the three-terminal memristor weight parameter. Therefore, the memristor is added as a feedback term to Equation (12). The divergence of Equation (17) is

(18) $$\nabla \cdot \dot{x}=\sum_{i=1}^{5}\frac{\partial f_{x_{i}}}{\partial x_{i}}=0.$$

which means the phase volume of the system (17) is still conservative. 

Now, we test whether the Hamiltonian energy is still conservative. The generalized Hamiltonian form was used [33]. We consider the input of the fifth term as a non-conservative force, and get

(19) $$\dot{x}=M(x)\nabla H(x),$$

where

(20) $$M(x)\nabla H(x)=J_{m}(x)\nabla H(x)+R(x)\nabla H(x)=f_{c}(x)+f_{d}(x),$$

with new Hamiltonian

(21) $$H=\frac{1}{2}(x_{1}^{2}+x_{2}^{2}+x_{3}^{2}+x_{4}^{2}+x_{5}^{2}),$$

and

$$\begin{array}{l} J_{m}(x)=\left[\begin{array}{cccc} \begin{array}{cc} \begin{array}{c} 0\\ -0.5x_{3}\\ -0.5x_{2}\\ \begin{array}{c} -\gamma (\alpha +\beta x_{5}^{2})\\ 0 \end{array} \end{array} & \begin{array}{c} 0.5x_{3}\\ 0\\ 1.5\left(x_{1}-x_{4}\right)\\ \begin{array}{c} 0.5x_{3}\\ 0 \end{array} \end{array} \end{array} & \begin{array}{c} 0.5x_{2}\\ 1.5\left(x_{4}-x_{1}\right)\\ 0\\ \begin{array}{c} 0.5x_{2}\\ 0 \end{array} \end{array} & \begin{array}{c} \gamma (\alpha +\beta x_{5}^{2})\\ -0.5x_{3}\\ -0.5x_{2}\\ \begin{array}{c} 0\\ 0 \end{array} \end{array} & \begin{array}{c} 0\\ 0\\ 0\\ \begin{array}{c} 0\\ 0 \end{array} \end{array} \end{array}\right],\\ R(x)=\left[\begin{array}{ccccc} 0 & 0 & 0 & 0 & 0\\ 0 & 0 & 0 & 0 & 0\\ 0 & 0 & 0 & 0 & 0\\ 0 & 0 & 0 & 0 & 0\\ 0 & 0 & 0 & 0 & \frac{x_{1}x_{4}}{x_{5}} \end{array}\right],f_{c}(x)=\left(\begin{array}{c} \begin{array}{c} x_{2}x_{3}+\gamma (\alpha +\beta x_{5}^{2})x_{4}\\ -2x_{1}x_{3}+x_{3}x_{4} \end{array}\\ x_{1}x_{2}-2x_{2}x_{4}\\ x_{2}x_{3}-\gamma (\alpha +\beta x_{5}^{2})x_{1}\\ 0 \end{array}\right),f_{d}(x)=\left(\begin{array}{c} \begin{array}{c} 0\\ 0 \end{array}\\ 0\\ 0\\ x_{1}x_{4} \end{array}\right). \end{array}$$

M(x) is no longer skew-symmetric but is decomposed into the sum of a skew-symmetric matrix J(x) and a symmetric matrix R(x). The total force exerted on the system is non-conservative. Differentiating the Hamilton function, we get

(22) $$\dot{H}=(x_{1}{\dot{x}}_{1}+x_{2}{\dot{x}}_{2}+x_{3}{\dot{x}}_{3}+x_{4}{\dot{x}}_{4}+x_{5}{\dot{x}}_{5})=x_{1}x_{4}x_{5}=\nabla H^{T}f_{d}(x)=x_{1}x_{4}x_{5},$$

which indicates Hamiltonian energy function is no longer conservative. We divide H of Equation (21) into

(23) $$H=H_{c}+H_{n},$$

with

$$H_{c}=\frac{1}{2}(x_{1}^{2}+x_{2}^{2}+x_{3}^{2}+x_{4}^{2}),H_{n}=\frac{1}{2}x_{5}^{2}.$$

It can be proved that ${\dot{H}}_{c}=0$, but ${\dot{H}}_{n}=x_{1}x_{4}x_{5}$. Therefore, $H_{c}$ represents the conservative energy of the system, and $H_{n}$ is the non-conservative part. According to [17], there are two categories of conservative systems: the Hamiltonian conservative chaotic system, in which both the volume and Hamiltonian of the system are constant, and the non-Hamiltonian conservative chaotic system, in which only the phase volume is conservative. Thus, the proposed three-terminal-memristor-based system (i.e., Equation (17)) is a typical non-Hamiltonian conservative chaotic system. 

## 4. Equilibria and Their Stability of Three-Terminal Memristor Conservative System

The equilibria point plays an essential role in analyzing the system’s properties and we examined whether it meets the requirements of the conservative chaotic system. For a conservative system, only saddles and centers exist. There are no stable or unstable nodes and foci to exist in a conservative system. Equation (17) can be rewritten as

(24) $$\begin{array}{l} {\dot{x}}_{1}=x_{2}x_{3}+\alpha \gamma x_{4}+\beta \gamma x_{4}x_{5}^{2},\\ {\dot{x}}_{2}=-2x_{1}x_{3}+x_{3}x_{4},\\ {\dot{x}}_{3}=x_{1}x_{2}-2x_{2}x_{4},\\ {\dot{x}}_{4}=x_{2}x_{3}-\alpha \gamma x_{1}-\beta \gamma x_{1}x_{5}^{2},\\ {\dot{x}}_{5}=x_{1}x_{4}. \end{array}$$

Setting the left of Equation (24) equal to 0, we can get three cases:

Case 1: $x_{1}=0,x_{4}=0$, we can get $x_{2}x_{3}=0,x_{5}\in R$. This case has three sub-cases as follows:

  Case 1.1:

    $x_{1}=0,x_{2}=0,x_{3}=0,x_{4}=0,x_{5}=x_{5},$

  Case 1.2:

    $x_{1}=0,x_{2}=0,x_{3}\neq 0,x_{4}=0,x_{5}=x_{5},$

  Case 1.3:

    $x_{1}=0,x_{2}=0,x_{3}\neq 0,x_{4}=0,x_{5}=x_{5},$

Case 2:

 $x_{1}\neq 0,x_{4}=0.$

Case 3:

 $x_{1}=0,x_{4}\neq 0.$

In Case 2, we derive $x_{2}=x_{3}=0$, but parameters α,β>0 and the weight parameter γ≠0, $\gamma (\alpha +\beta x_{5}^{2})x_{1}=0$ holds. Since $x_{1}\neq 0$, we have $\gamma \left(\alpha +\beta x_{5}^{2}\right)=0$ which contradicts the premise. Therefore, Case 2 does not hold. Case 3 has the same problem as Case 2. Therefore, from Case 1, the system has line equilibria $E_{5}={\left[0,0,0,0,x_{5}\right]}^{T}$, plane equilibria $E_{3,5}={\left[0,0,x_{3},0,x_{5}\right]}^{T}$, and $E_{2,5}={\left[0,x_{2},0,0,x_{5}\right]}^{T}$. The Jacobi matrix of the system is

(25) $$J=\left[\begin{array}{cccc} \begin{array}{cc} \begin{array}{c} 0\\ -2x_{3}^{*}\\ x_{2}^{*}\\ \begin{array}{c} -\gamma (\alpha +\beta x_{5}^{*}{}^{2})\\ x_{4}^{*} \end{array} \end{array} & \begin{array}{c} x_{3}^{*}\\ 0\\ x_{1}^{*}-2x_{4}^{*}\\ \begin{array}{c} x_{3}^{*}\\ 0 \end{array} \end{array} \end{array} & \begin{array}{c} x_{2}^{*}\\ x_{4}^{*}-2x_{1}^{*}\\ 0\\ \begin{array}{c} x_{2}^{*}\\ 0 \end{array} \end{array} & \begin{array}{c} \gamma (\alpha +\beta x_{5}^{*}{}^{2})\\ x_{3}^{*}\\ -2x_{2}^{*}\\ \begin{array}{c} 0\\ x_{1}^{*} \end{array} \end{array} & \begin{array}{c} 2\gamma \beta x_{4}^{*}x_{5}^{*}\\ 0\\ 0\\ \begin{array}{c} -2\gamma \beta x_{1}^{*}x_{5}^{*}\\ 0 \end{array} \end{array} \end{array}\right].$$

By substituting $E_{5}$ into the characteristic equation, we find the eigenvalues of $E_{5}$ as

(26) $$E_{5}=(0,0,0,0,x_{5})\to \lambda =(0,0,0,-\gamma j(\beta x_{5}^{2}+\alpha ),\gamma j(\beta x_{5}^{2}+\alpha )).$$

Hence, line equilibria $E_{5}$ are centers. 

For the plane equilibrium $E_{3,5}$, we find the eigenvalues as

(27) $$E_{3,5}=(0,0,x_{3},0,x_{5})\to \lambda =(0,0,a_{4},-\frac{1}{2}a_{4}-j\frac{\sqrt{3}}{2}a_{5},-\frac{1}{2}a_{4}+j\frac{\sqrt{3}}{2}a_{5}),$$

where

$$\begin{array}{l} a_{1}=\frac{\alpha^{2}\gamma^{2}}{3}+\frac{2\alpha \beta \gamma^{2}x_{5}^{*}{}^{2}}{3}+\frac{\beta^{2}\gamma^{2}x_{5}^{*}{}^{4}}{3}+\frac{x_{3}^{*}{}^{2}}{3},\\ a_{2}=\frac{3\beta \gamma x_{3}^{*}{}^{2}x_{5}^{*}{}^{2}}{2}+\frac{3\alpha \gamma x_{3}^{*}{}^{2}}{2},\\ a_{3}=\sqrt[{3}]{\sqrt{a_{1}^{3}+a_{2}^{2}}-a_{2}},\\ a_{4}=a_{3}-\frac{a_{1}}{a_{3}},\\ a_{5}=2a_{3}-a_{4}. \end{array}$$

where j is the imaginary unit. If $a_{4}=0$ holds, $E_{3,5}$ must be centers from Equation (24). If $a_{4}\neq 0$, we can find out if $a_{4}>0$, $\lambda_{3}$ is a positive real number and the real parts of $\lambda_{4},\lambda_{5}$ must be negative. If $a_{4}>0$, $\lambda_{3}$ is a negative real number and the real parts of $\lambda_{4},\lambda_{5}$ must be positive. Therefore, if $a_{4}\neq 0$, $E_{3,5}$ must be saddles. Likewise, plane equilibrium $E_{2,5}$ has the same properties as $E_{3,5}$. In sum, both $E_{2,5}$ and $E_{3,5}$ are either natural elliptic (centers) or saddles. Thus, from the perspective of these equilibria, the system fully meets the characteristics that the conservative system in phase space only has either saddle or center [17,18,19].

## 5. Dynamical Analysis of Three-Terminal Memristor Conservative Chaotic System

### 5.1. Memristor Effect in Chaos Generation 

**Figure 6:**
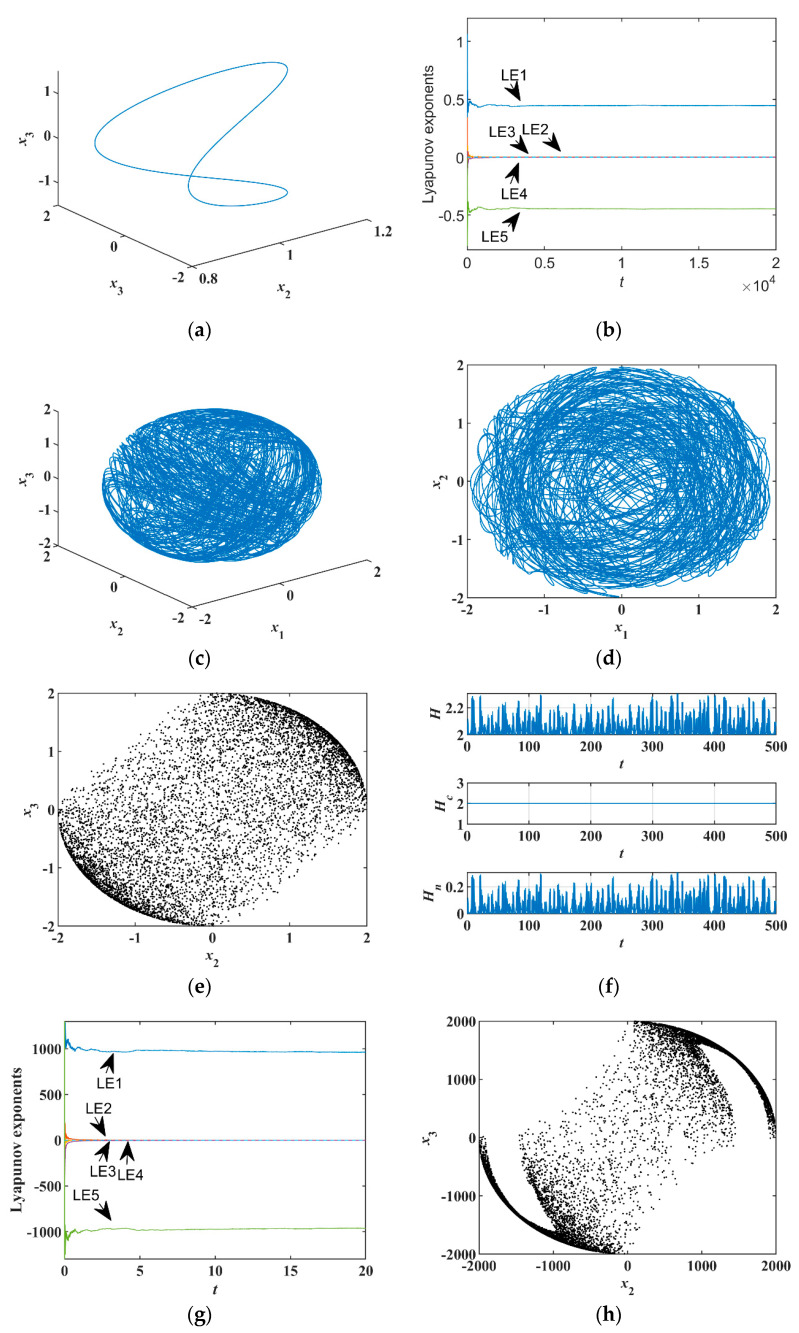
Numerical characteristics of the system of Equation (19): (**a**) 3D view of $x_{1}-x_{2}-x_{3}$ with γ=0, (**b**) Lyapunov exponents with γ=1, (**c**) 3D view of $x_{1}-x_{2}-x_{3}$ with γ=1, (**d**) phase portrait of $x_{1}-x_{2}$ with γ=1, (**e**) Poincaré map of $x_{2}-x_{3}$ with $x_{1}=0$, (**f**) Hamiltonian energy, (**g**) Lyapunov exponent (*LE*s) with large coefficients, and (**h**) Poincaré map with large coefficients.

For system (19), take initial values ${\left[x_{10},\;x_{20},\;x_{30},\;x_{40},\;x_{50}\right]}^{T}={\left[1,\;1,\;-1,\;-1,\;0\right]}^{T}$, parameters ${\left[\alpha ,\;\beta ,\;\gamma \right]}^{T}={\left[1,\;1,\;0\right]}^{T}$, and sampling time T=0.001 s. Since γ=0, the outputs of the three-terminal memristor do not affect the system. According to the analysis of Equation (10), it produces periodic orbit, as shown in Figure 6a.

Now fixing α=β=γ=1 and initial values as above, the orbits of different phase spaces are shown in Figure 6c,d. Therefore, the three-terminal-memristor-based conservative system produces chaos, which is called the three-terminal-memristor-based conservative chaotic system. The Poincaré map from Figure 6e with $x_{1}=0$ shows the orbits are chaotic and do not form a chaotic attractor. The chaotic attractor of a dissipative system has several little branches of hair-like Poincaré map because of the fractal dimension, but this memristor-based conservative chaos has a wide-banding Poincaré map that almost evenly fills the space initially occupied. This is because it has an integer dimension. The ergodicity of the memristor-based conservative chaotic system is much better than general dissipative chaotic systems, which is beneficial in chaos-based encryption.

The *LE*s $L_{1,2,3,4,5}={\left[0.4446,\;0.0003,\;0.0000,\;-0.0003,\;-0.4446\right]}^{T}$ in Figure 6b, indicating the sum of the *LE*s is zero. From Ref. [20], the Kaplan-Yorke Lyapunov dimension is

(28) $$L_{KY}=4+\left(L_{1}+L_{2}+L_{3}+L_{4}\right)/\left|L_{5}\right|=5.$$

The integer result proves the conservativeness of the system. From Figure 6f, $H_{c}$ is constant, indicating this part is conservative; however, the total Hamiltonian of the system H changes, which is caused by the non-conservative part $H_{n}$. Thus, we confirmed the system is a typical non-Hamiltonian conservative chaotic system. 

In this case, the maximum *LE* value, $L_{1}=0.4446$, is small, and the Poincaré map area is not large enough, although it fills evenly. Normally, in chaos-based encryption, the larger maximum *LE* value, the higher-order in pseudo-randomness of the chaos generator, the better in security, the more difficult to break. Besides, the larger area of Poincaré map, the more choices in the selection of pseudo-randomness. To enhance the maximum *LE*, many references took a trial-and-error method through bifurcation; we took a mechanism way in this paper. Provided that the frequency and energy of the system are increased, the positive Lyapunov exponent can be increased. Therefore, we adjusted the five initial values and four parameters according to their functions in frequency and energy. According to the 4D Euler equation, increasing $\pi_{i}$ can speed up the operating frequency of the system, and increasing the initial values can store the kinetic energy. The initial values also determine the spatial magnitude of orbits and the area of the Poincaré map. Initial values ${\left[x\right]}^{T}={\left[1000,\;1000,\;-1000,\;-1000,\;0\right]}^{T}$ and ${\left[\pi_{1},\;\pi_{2},\;\pi_{3},\;\pi_{4}\right]}^{T}={\left[100,\;150,\;200,\;250\right]}^{T}$, and sampling time $T=10^{-7}$ s, were chosen to calculate Lyapunov exponents using the Wolf method [34]. As shown in Figure 6g,h, the maximum of the *LE*s $LE_{1}=0.9631\times 10^{3}$. The maximum of the *LE*s is far greater than those of the most chaotic systems; it can be observed from the Poincaré map with $x_{1}=0$ that the space of the orbits is quite large, and the frequency is very high, which can be tested by frequency spectrum (the figure is omitted). Usually, the maximum *LE* cannot be adjusted too large through mechanical analysis. To increase the *LE*, the scaling time can be adjusted; however, this proposed memristor-based chaotic system can produce exceptionally large maximum *LE* through the adjustments of parameter and initial, which is based on the physical mechanism. The large *LE* and magnitude of Poincaré map are greatly helpful in chaos-based encryption. 

### 5.2. Dynamical Analysis with Different Initial Conditions of $H_{c}$

We have analyzed the three-terminal memristor excitation of chaos, so how does Hamiltonian affect system dynamics? Fixing parameter ${\left[\alpha ,\;\beta ,\;\gamma \right]}^{T}={\left[1,\;1,\;1\right]}^{T}$, the initial value determines $H_{0}$ from Equation (29).

(29) $$H_{0}\geq H_{c0}=\frac{1}{2}(x_{10}^{2}+x_{20}^{2}+x_{30}^{2}+x_{40}^{2})$$

**Figure 7:**
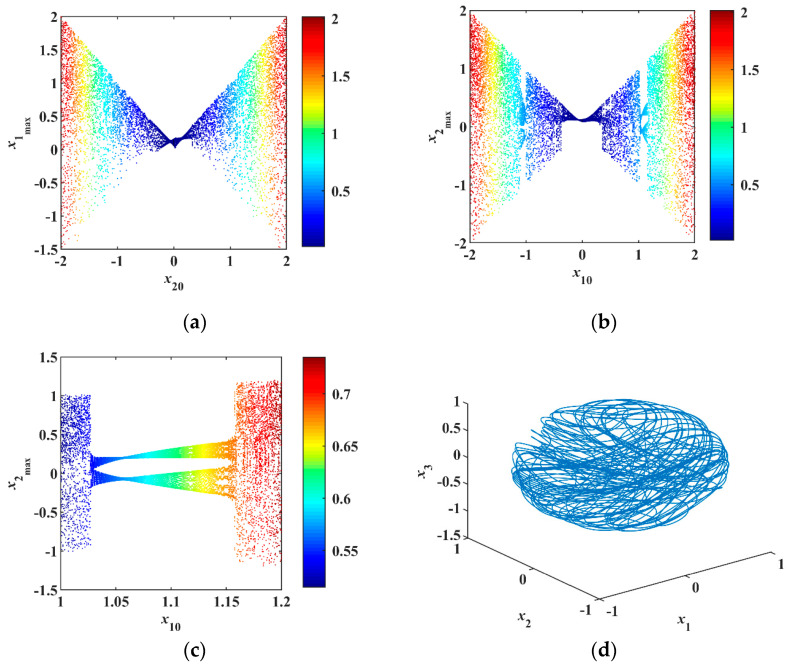

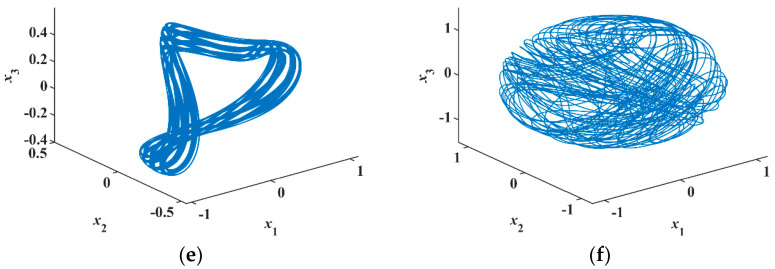
Dynamical analysis with different initial conditions: (**a**) Bifurcation within initial $x_{20}\in \left[-2,2\right]$, (**b**) bifurcation within initial $x_{10}=\left[-2,2\right]$, (**c**) bifurcation within initial $x_{10}\in \left[1,1.2\right]$, (**d**) chaotic orbits with $x_{10}=1$, (**e**) quasiperiodic orbits with $x_{10}=1.1$, (**f**) chaotic orbits with $x_{10}=1.2$.

Choose the initial value $x_{0}={\left[0.1,\;x_{20},\;0.1,\;0.1,\;0\right]}^{T}$ with $x_{20}\in \left[-2,\;2\right]$, $x_{0}={\left[x_{10},\;0.1,\;0.1,\;0.1,\;0\right]}^{T}$ with $x_{10}\in \left[-2,\;2\right]$. The bifurcation diagrams are plotted by removing 80% of transients. Both initials correspond to the Hamiltonian $H_{0}\in \left[0.015,\;2.015\right]$. Figure 7a,b are the bifurcation diagrams based on the change of initials. The stripe colors represent Hamiltonian $H_{0}$ marked by the color bar. The low Hamiltonian energy (dark blue and blue) represents quasiperiodic orbits, and the high Hamiltonian energy (light blue, yellow, and red) represents chaos.

Figure 7c is the zoom of Figure 7b within $x_{10}\in \left[1,\;1.2\right]$. There are some bifurcations, for instance, when $x_{10}=1$ and $x_{10}=1.2$, the system produces chaotic orbits, as shown in Figure 7d,f. However, it also generates the periodic orbits, period-2 orbits, and quasiperiodic orbits with $x_{10}=1.1$, as shown in Figure 7e.

### 5.3. Dynamical Analysis with Fixed $H_{c}$ and Varied $H_{n}$

Although the non-conservative part $H_{n}$ changes with time at the rate of Equation (22), in Section 5.1 and Section 5.2, the initial value of $x_{50}\neq 0$ is crucial. When it is set to zero, the influence of three-terminal memristor variable is reduced as much as possible from Equation (23). In this section, the four initial values ${\left[x_{10},\;x_{20},\;x_{30},\;x_{40}\right]}^{T}$ corresponding to the conservative part $H_{c0}$ being fixed, we analyze how the initial value $x_{50}$ corresponding to the $H_{n0}$ effects of the system. Fix the initial values ${\left[x_{10},\;x_{20},\;x_{30},\;x_{40}\right]}^{T}={\left[0.1,\;0.1,\;0.1,\;0.1\right]}^{T}$ with $H_{c0}=0.02$ and ${\left[x_{10},x_{20},\;x_{30},\;x_{40}\right]}^{T}={\left[1,\;1,\;1,\;1\right]}^{T}$ with $H_{c0}=2$, and

(30) $$H_{n0}=\frac{1}{2}x_{50}^{2}.$$

**Figure 8:**
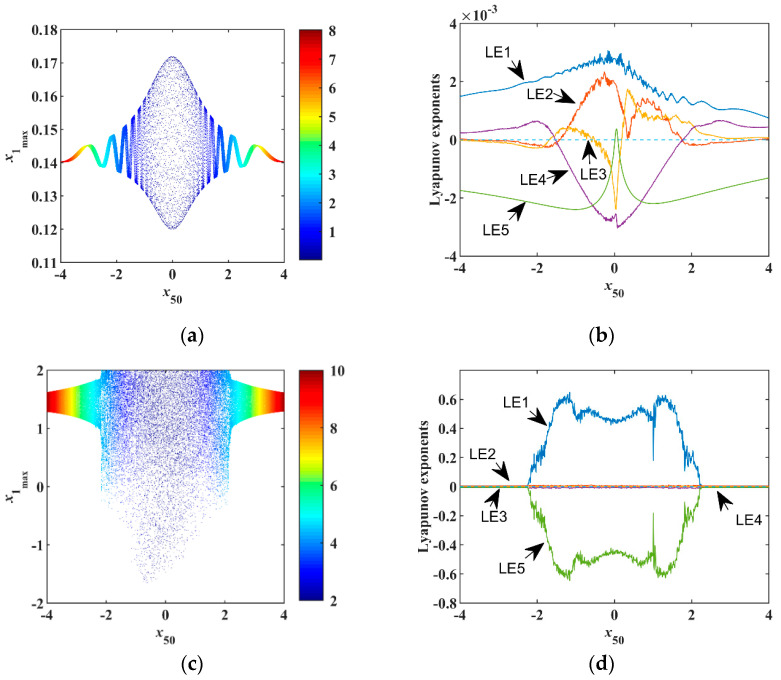
Dynamical analysis with fixed $H_{c0}$, (**a**) Bifurcation of $x_{50}$ with $H_{c0}=0.02$, (**b**) *LE*s of $x_{50}$ with $H_{c0}=0.02$, (**c**) bifurcation of $x_{50}$ with $H_{c0}=2$, (**d**) *LE*s of $x_{50}$ with $H_{c0}=2$.

The low $H_{c0}$ energy determines that the system generates quasiperiodic orbits [Figure 8a] and the high $H_{c0}$ energy makes chaos [Figure 8c], which corresponds to the results given in Section 5.1 and Section 5.2. Regardless of the value of conservative Hamiltonian $H_{c0}$, $x_{50}$ promotes the chaotic degree of the system within a range. Beyond this range, it suppresses the system’s dynamics and transits from chaotic motion to quasiperiodic motion. As the Hamiltonian level increases, the range becomes wider, as shown in Figure 8b,d.

## 6. Circuit Implementation

There are three methods for the simulation of nonlinear systems: chaotic system simulation circuit, FPGA (Field Programmable Gate Array), and computer numerical simulation. For the most dissipative systems, the required accuracy is not high, so all three simulation methods can be implemented. However, for conservative systems, the required accuracy is very high, especially FPGA and numerical simulation. Even if the accuracy is high, there still are problems such as algorithms, sampling, discretization, and the number of bits of computer operations causing errors. The conservative system divergence is zero, so a little bit of error will make the divergence either larger than zero leading to instability, or less than zero leading to shrinking. Both situations cause the conservative chaotic phase diagram to disappear. The hardware implementation is challenging. Therefore, for conservative systems, analog circuit simulation is indispensable because it is a real analog simulation.

**Figure 9:**
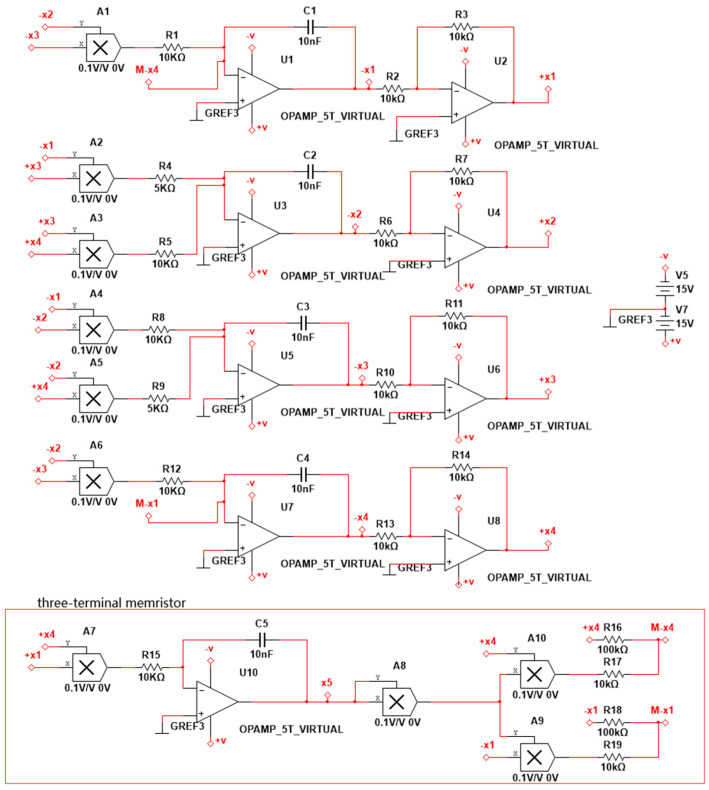
Circuit implementation of system Equation (15), with electronic parameters: R1,R2,R3,R5,R6,R7,R8,R10,R11,R12,R13,R14,R15,R17,R19=10 KΩ; R4,R9=5 KΩ; R16,R18=100 KΩ; C1,C2,C3,C4,C5=10 nF.

As shown in Figure 9, the system is implemented by an analog electronic circuit without scale transformation. A total of 10 analog multipliers accomplish seven quadratic terms and two cubic terms in the system. Besides, there are five integrators and four inverters, which are composed of some capacitors and resistors. The three-terminal memristor is constructed at the bottom of Figure 9.

**Figure 10:**
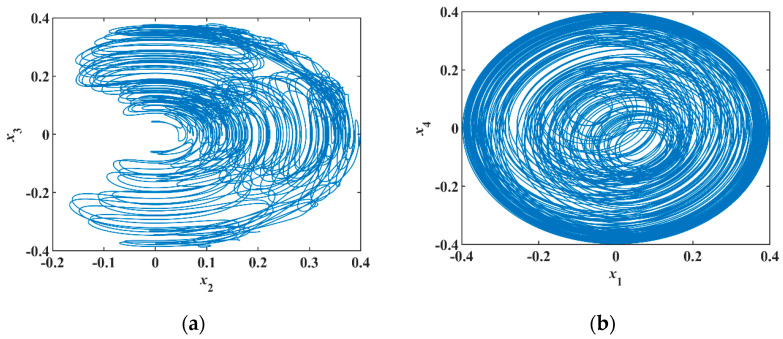
Phase portrait of numerical simulation: (**a**) Phase portrait of $x_{2}-x_{3}$, (**b**) phase portrait of $x_{1}-x_{4}$.

Selecting the initial values ${\left[x_{0}\right]}^{T}={\left[0.2,\;0.2,\;0.2,\;0.2,\;0\right]}^{T}$ and parameters ${\left[\alpha ,\;\beta ,\;\gamma \right]}^{T}={\left[1,\;0.1,\;1\right]}^{T}$, the phase portraits of numerical simulation are shown in Figure 10a,b. In the analog circuit, the chips have a slight voltage deviation that corresponds to a small initial value, so there is no need to add the initial voltage between the capacitor’s two pins.

**Figure 11:**
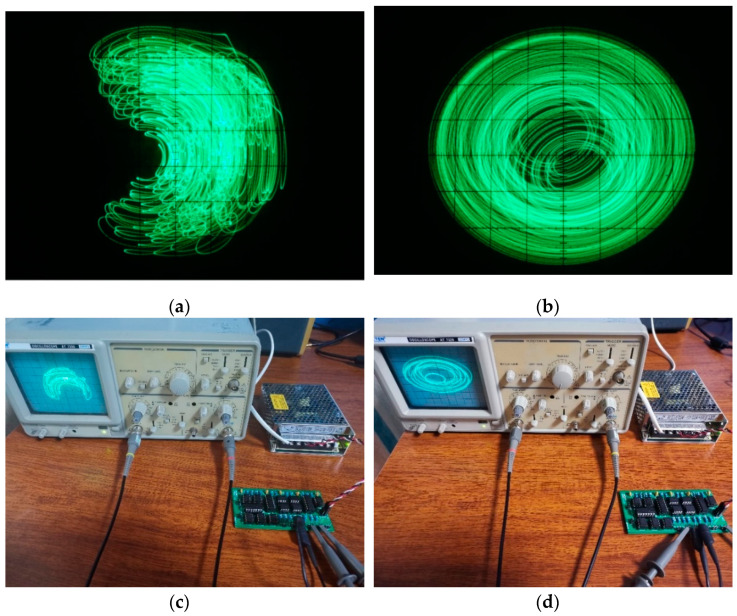
Implementation of circuit: (**a**) Phase portrait of $x_{2}-x_{3}$, (**b**) phase portrait of $x_{1}-x_{4}$, (**c**) implementation of the circuit, (**d**) implementation of the circuit.

Figure 11a,b showing different phase diagrams are consistent with the results of a numerical simulation showing in Figure 10a,b. It is verified that the proposed system produces conservative chaos.

## 7. Conclusions

This paper proposed a non-Hamiltonian conservative chaotic system by integrating three-terminal memristor and 4D Euler equations. The dual-output pins of the three-terminal memristor, satisfying the nature of the skew-symmetric matrix. The system has generalized Hamiltonian; the conservation of the 4D Euler equation has been preserved. The characteristics of either centers or saddles of the equilibrias of the system proved the conservation property. Chaotic dynamics have been revealed by varying the weight parameter of the three-terminal memristor. Changing the initial Hamiltonian of the system will produce rich dynamics, which provides the way of producing quasiperiodic orbit and chaos. The routes and mechanisms from quasiperiodic orbit to chaos have been provided through energy bifurcation. With different initial Hamiltonian levels, the system will have different dynamic ranges. Using energy and frequency adjustment, instead of trial-and-error, the system produced the huge *LE*s, which is more suitable for encryption than other chaotic systems. The analog circuit of the system was built physically, which confirmed the chaotic existence of the system, and combined the three-terminal memristor and 4D Euler equation successfully. By changing the different types of memristors, modifying the dual outputs, and embedding it into the 4D Euler equation, keeping the conservative part skew-symmetric nature, chaos can be generated under reasonable parameters.

## Data Availability

Not applicable.
